# The Significance of Religion and Spirituality Among Turkish Women Surviving Breast Cancer Without Treatment: A Qualitative Study

**DOI:** 10.1007/s10943-024-02039-y

**Published:** 2024-04-15

**Authors:** Yasemin Özyer Güvener

**Affiliations:** Faculty of Health Sciences, Sinop University, Sinop, Turkey

**Keywords:** Breast cancer, Nursing theory, Religion, Spirituality

## Abstract

The health of breast cancer survivors is a global concern. It is crucial to adopt a holistic approach when understanding their journey from illness to wellness in order to ensure that the transition is as smooth as possible. This study focused on the experiences of Muslim women who had overcome breast cancer and were adapting to life post-treatment. Snowball sampling was used to select the participants for this qualitative study. Fifteen women who had successfully completed breast cancer treatment and been declared cancer-free were interviewed. These interviews were semi-structured, using open-ended questions to explore their experiences in-depth. The interviews were conducted by phone, and the data were analyzed using qualitative content analysis. The study identified four main themes, nine subthemes, and 41 codes. The main themes were as follows: (a) a life changed by cancer and the difficulties encountered; (b) transition from active treatment to treatment-free living; (c) coping mechanisms; and (d) future hopes and expectations. The findings highlighted the survivors’ desire to move past their experience of cancer and normalize their lives, as well as emphasizing their need for support. The participants shared detailed accounts of their journey, the obstacles they encountered during this transition, and the critical role of religion and spirituality in overcoming these challenges. Understanding and effectively managing the experiences of women after breast cancer treatment is vital not only for improving survival rates but also for facilitating their healing process.

## Introduction

Breast cancer is the most prevalent type of cancer among women globally. One in every eight diagnoses of cancer is for breast cancer, with 2.3 million new cases in 2021 affecting both sexes (Sung et al., 2021). Accounting for a quarter of all female cancer cases, breast cancer was the most frequently diagnosed cancer in women in 2020. The burden caused by breast cancer is on the rise worldwide, especially in developing countries (Arnold et al., 2022; Heer et al., 2020). The diagnosis and treatment of cancer impact individuals in numerous ways. For instance, the fear of recurrence, the financial strain of cancer treatment, and a lack of social support are significant stressors for those dealing with breast cancer (Hu et al., 2021).

The impact of breast cancer diagnosis and treatment extends beyond the physical aspects and is influenced by patients’ cultural and religious beliefs, as well as their social lives (Sarang et al., 2023). While treatments aimed at preventing the disease have reduced mortality rates, the effects of both diagnosis and treatment can permeate all facets of a patient’s life (Ciria-Suarez et al., 2021). Following the completion of treatment, patients often face challenges such as diminished physical and mental capabilities, fear of the cancer returning, and alterations in their professional lives (Ciria-Suarez et al., 2021; Miroševič et al., 2019).

Qualitative research regarding breast cancer frequently explores themes like body image, social support systems, and reintegration into everyday life for survivors. Despite this, there is a noticeable lack of studies that specifically investigate the experiences of women with breast cancer from diagnosis through to recovery (Adam & Koranteng, 2020; Faccio et al., 2020). Gaining a deeper understanding of the different phases patients go through during this journey, and adopting a holistic viewpoint, can significantly enhance the care provided to these individuals (Ciria-Suarez et al., 2021).

Living with breast cancer often brings a range of physical, emotional, and psychological challenges due to the extended nature of treatment. Many patients describe the treatment period as highly traumatic and emotionally taxing (Lambert et al., 2020). During this challenging time, women frequently turn to religious practices and spirituality for support (Sarang et al., 2023). Various studies have explored coping strategies among breast cancer patients. For instance, in Iran, it was found that religious beliefs were a significant element in the coping strategies of many breast cancer patients (Hu et al., 2021; Sarang et al., 2023).

Common ways of coping include prayer, seeking social support, avoiding negative situations, and maintaining a strong will to live. Research has also indicated that these mechanisms play a crucial role in adapting to breast cancer and can influence the disease’s progression (Hu et al., 2021). Women often lean on their spirituality and religious beliefs to navigate the psychosocial challenges encountered during and after their cancer treatment.

Spirituality and religious coping tend to be more prevalent among breast cancer patients than in other groups. As such, spirituality and religion constitute an essential aspect of psychosocial care in this context (Ko et al., 2023). Successfully transitioning from active treatment to a treatment-free period is crucial, not only for enhancing patients’ quality of life but also for improving their chances of survival (Lambert et al., 2020).

Transition theory, as outlined by Meleis, offers a comprehensive framework for understanding and predicting specific life events (Meleis et al., 2000). This theoretical approach can inform research, leading to more effective and more positive outcomes. It also provides a generalizable model to understand the transition experiences of breast cancer patients, guiding both research and practice in this area (Chao et al., 2020; Meleis et al., 2000).

The present study aimed to understand the needs and experiences of patients after they had completed active breast cancer treatment, focusing particularly on their experiences during cancer survivorship. Utilizing Meleis’s transition theory as the guiding theoretical framework, this study was conducted to determine the importance of religion and spirituality in transition experience of female patients who have survived breast cancer to a treatment free life.

### Theoretical Framework

This study explores the journey of breast cancer patients as they navigated from a state of illness to one of health. It is framed within Meleis’s transition theory, which posits that the essence of a transition lies in the factors that drive it. While change is an external event, transition is an internal process (Meleis, 2015). It involves moving from one stable state to another and is inherently dynamic (Meleis, 2015; Meleis et al., 2000).

This process is influenced by various enabling and hindering factors at the personal, community, and societal levels (Meleis, 2010). The effectiveness of a transition is determined by multiple elements, including individual and environmental factors, a person’s readiness, their level of knowledge, and the meaning they attribute to the transition (Aydin & Bulut, 2022; Meleis et al., 2000). Transition theory is utilized to elucidate the experiences of individuals who are navigating, enduring, and adapting to changes that require them to develop new behaviors, emotions, skills, and roles (Meleis, 2015).

This theory posits that individuals undergoing a transition will encounter personal uncertainty and change, leading to a redefinition of their identity (Meleis, 2010). Meleis (2010) suggests that a successful transition is marked by enhanced self-esteem, behavioral changes, and the development of a positive perspective. The theory encompasses four interconnected main concepts: (a) the nature of the transition; (b) factors that facilitate and inhibit the transition; (c) response patterns to the transition; and (d) nursing therapeutics.

Through the lens of Meleis’s theory, it becomes apparent that personal, familial, and societal factors are interconnected, each playing a role in shaping the transition experience. For breast cancer survivors, the transition process is prolonged and persistently uncertain. This uncertainty is multifaceted, influencing the physical, psychological, and emotional well-being of patients, as well as their interpersonal and social interactions. In the present study, Meleis’s transition theory informed the development of the preliminary coding list used during the creation of interview questions and the subsequent data analysis phase.

## Methods

### Study Design

This study utilized an exploratory qualitative design. The qualitative methodology is especially suited for an in-depth understanding of an individual’s experience in both its complete form and its individual elements (Creswell, 2013).

The sample of the study comprised female individuals who had received treatment in a hospital in northern Türkiye and had completed their treatment and then returned to their normal daily lives. To gather participants, the study employed the snowball sampling method. During the interviews, the researcher used a semi-structured interview format, while also incorporating specific exploratory and interpretive questions to ensure diversity and depth in the responses. The data were collected and reported according to the checklist created by the Consolidated Criteria for Qualitative Studies (COREQ) (Tong et al., 2007).

### Participants and Setting

The study encompassed women who were over 18 years old, had completed their breast cancer treatment, were free of metastasis or psychiatric disorders, and were able to read and write. Data collection occurred between May and August 2023. The participants were contacted by telephone, and each interview lasted between 30 and 45 min. The interview questions were developed on the basis of Meleis’s transition theory (Meleis, 2010), focusing on the individuals’ experiences post-breast cancer treatment and the key factors influencing these experiences.

Examples of these questions include: “Can you share your experiences of transitioning to treatment-free living after your breast cancer treatment was completed?” and “How did religion and spirituality play a role in your life during the transition process?” A total of 15 women volunteered for the study. The study concluded when responses began to be repeated, indicating that data saturation had been achieved. The interviews were conducted by the lead researcher, who had prior experience in qualitative research, using a pre-designed interview form.

### Data Collection, Analyses and Synthesis

To help answer the research questions, all data were carefully read, transcribed, organized, and labeled with specific codes. These codes were then grouped according to their differences and similarities, forming themes that connected the underlying meanings of the categories. Themes, subthemes, and additional codes were developed to assist in synthesizing the data and interpreting the findings.

The data analyzed and the materials were reviewed repeatedly to ensure accuracy. Reliability was also secured by thoroughly validating each code used in the analysis. This process was conducted in alignment with the standards for qualitative content analysis. The data were analyzed using qualitative content analysis and statistically analyzed using the MAXQDA 20 software.

## Results

The sociodemographic characteristics of the participants are presented in Table 1.

**Table 1:** Socio-demographic characteristics of the participants (*n*:15)

Participant	Age	Marital status	Education	Occupation	Clinical stage	Having social security	Religion
P1	46	Married	High School	Housewife	Stage 3	Yes	Muslim
P2	33	Single	University	Self-employed	Stage 1	Yes	Muslim
P3	55	Married	Primary school	Housewife	Stage 1	Yes	Muslim
P4	42	Single	University	Self-employed	Stage 2	Yes	Muslim
P5	31	Married	University	Housewife	Stage 3	Yes	Muslim
P6	39	Single	University	Teacher	Stage 3	Yes	Muslim
P7	57	Single	Primary school	Housewife	Stage 3	Yes	Muslim
P8	48	Married	Primary school	Housewife	Stage 3	Yes	Muslim
P9	58	Married	Primary school	Housewife	Stage 3	Yes	Muslim
P10	55	Married	Primary school	Self-employed	Stage 1	Yes	Muslim
P11	50	Single	University	Self- employed	Stage 2	Yes	Muslim
P 12	52	Married	Primary school	Housewife	Stage 3	Yes	Muslim
P 13	27	Married	High school	Housewife	Stage 1	Yes	Muslim
P 14	53	Married	Primary school	Self-employed	Stage 2	Yes	Muslim
P 15	57	Married	University	Government employed	Stage 3	Yes	Muslim

Following the interviews and subsequent analysis, four main themes, nine subthemes, and 41 codes were identified. The themes were as follows: (a) a life changed by cancer and the difficulties encountered; (b) transition from active treatment to treatment-free living; (c) coping mechanisms; and (d) future hopes and expectations. The details of these themes are outlined in Table 2.

**Table 2:**
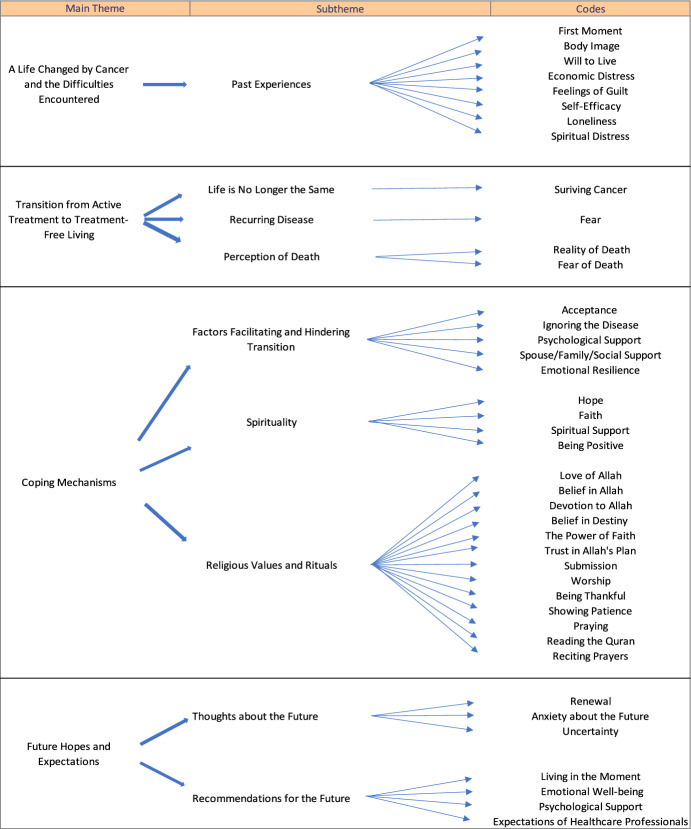
Themes related to the qualitative opinions of the participants (*n*: 15)

### Theme 1: A Life Changed by Cancer and the Difficulties Encountered

In the first theme, the participants discussed the changes that cancer had brought into their lives, particularly focusing on the difficulties they encountered during the treatment process. This discussion led to the development of a subtheme entitled “Past Experiences.” The challenges faced by the participants in their past encompassed economic, spiritual, psychological, and social changes. The theme was characterized by a range of concepts, including the emotions felt on first hearing the cancer diagnosis, spiritual distress, the will to live, body image concerns, feelings of guilt, self-sufficiency, and loneliness. For example, some participants expressed feelings of incompleteness related to physical changes, such as those experienced after breast surgery, and the impact of hair loss due to chemotherapy.

Some participants shared that a lack of financial support had forced them to sell their possessions, leading to a decrease in their self-esteem. They provided various accounts of these past experiences, as follows:

“You don’t think you can survive because you’re too young, you can’t help but be afraid. You ask yourself, ‘How can this be?’ During treatment, I thought about my hair first. I asked the nurse if my hair would fall out. I cried and cried in the hospital the day I learned I had cancer. I’ll never forget that day. I used to want to die when I had problems in the past. But when I got cancer, I said to Allah, ‘I want to live, I’m too young’.” (P2).

Some patients believed that breast cancer was a punishment from Allah and a test.

“I felt very alone during the time I was receiving chemotherapy. I told myself to stay strong; there's only Allah and you.” (P9).

“I believe Allah gave me this illness to teach me a lesson. It was a very difficult period for me, both materially and spiritually. I faced many challenges during both the chemotherapy and radiotherapy. I had to sell many things I owned.” (P1).

“My child was still in diapers at that time, and I couldn't take care of him. That was the hardest part for me, not being able to change my child's diapers.” (P5).

“There were days when I needed just 5 or 10 lira, and thinking back to those days makes me sad [participant was crying]. Thankfully, I'm in a better place now.” (P8).

### Theme 2: Transition from Active Treatment to Treatment-Free Living

In this theme, the participants shared their feelings and the thoughts that emerged after completing their treatment. This theme consisted of three subthemes. In the subtheme “Life is Different Now,” the majority of the women talked about their experiences of coping with cancer since their diagnosis. One of the participants’ statements was as follows:

The subtheme of “Fear of Disease Recurrence” stemmed from the fear of cancer returning.

“Do you know why I’m scared? It’s because of that red pill; it drives me crazy.” (P13).

“Of course, I keep thinking over and over, ‘Will it come back?’ It feels like I'll be stronger this time. I was caught off guard back then. I didn't know anything. Hopefully, I'll be stronger this time, but of course, I hope the disease doesn't come back.” (P8).

The subtheme of “Perception of Death” revolved around the possibility of dying and the participants’ fear of this. Most participants mentioned that they had accepted and internalized the concept of death. Some stated that they were not afraid of death and believed that it would happen when it was destined to. Some of the participants’ statements were as follows:

“You could be in perfect health, and you might have a traffic accident. You don't have any illnesses, but you can still die. So, you never know where death will come from. That's why I'm not afraid of death.” (P2).

“It does cross my mind. I wonder if it will recur. Personally, I am afraid of death. After all, we'll all leave this world someday, won't we? None of us are here forever.” (P11).

### Theme 3: Coping Mechanisms

This topic consisted of three subthemes: “Factors Facilitating and Hindering Transition”; “Spirituality”; and “Religious Values and Rituals.” The first subtheme encompassed concepts such as acceptance, denial of illness, psychological support, support from spouse, family, and social networks, and emotional resilience. Some of the participants’ statements were as follows:

“I mean, I can easily just say, ‘I have cancer, right?’ I’ve learned to live with it now, we’ve become buddies. There’s no escaping it, I’ve learned to coexist with it.” (P2).

“We never accepted this illness; we acted like it didn't exist, and we're still acting like it doesn't exist.” (P7).

“I know that I belong to a certain place, that I belong to oncology. But besides that, life goes on. It was like waking up from a nightmare that never really happened. I mean, nobody remembers it.” (P6).

“You're expecting attention from your spouse, you're expecting love, and you're upset because you can't take care of your child. It's really tough.” (P5).

“Throughout this journey, I’ve come to understand that it's important not to let anything get to you or make you upset. I’ve seen many spouses in similar situations, ones who don’t offer any support. For instance, my neighbor's husband got diagnosed with cancer, and he left her in that state.” (P10).

Spirituality was a recurring theme across all the conversations with the participants. After breast cancer treatment, having a sense of spirituality emerged as a source of support and well-being for the women, helping them reconnect with themselves. In many cases, the women experienced a deep form of spirituality, which allowed them to stay calm and balanced from the moment of diagnosis. It also served as a pillar that enabled them to maintain a semblance of normalcy in their lives.

Additionally, having an optimistic outlook was closely tied to hope and belief, and was seen as a positive coping mechanism during the challenging period of breast cancer diagnosis and treatment. Some of the women, when confronted with the disease, shifted their focus toward its spiritual meaning. Spirituality, for these women, became a crucial foundation and a motivating force to continue living. In their journey to overcome their illness, these women actively sought spiritual strength.

The subtheme of “Spirituality” encompassed hope, belief, and spiritual support. Some of the participants’ statements included:

"When my test results came in, everyone told me that they had been praying for me and that their prayers had come true. I'm someone who believes in the power of prayers." (P10).

The subtheme of “Religious Values and Rituals” encompassed concepts such as love for Allah, belief in Allah, devotion to Allah, belief in destiny, strength of one’s faith, trust, surrender, worship, gratitude, patience, prayer, reading the Quran, and saying prayers.

The feeling of having a connection with “the Creator” and being able to say prayers provided significant spiritual support to many women, leading to greater emotional strength. Some of the participants’ statements were as follows:

"What is hope? Well, I'm still hopeful, thank Allah. I prayed to Allah for help. I recited the Shahada. I constantly recited prayers and read the Quran. I expressed continuous gratitude. I prayed to Allah a lot, asking Him to forgive me and spare me for the sake of my children. When I prayed to Him, I felt a profound sense of well-being. I would advise women to be patient, patient, patient. They should trust in Allah and learn to be grateful in general." (P1).

"We sought refuge in Allah. We said, 'Lord, we came from You, and we will return to You. We have nowhere else to go.’ There's nothing we can do. Ultimately, I'm a Muslim, and I live by the principles of Islam. I find peace on my prayer rug." (P2).

"There's a concept called ‘tevekkül’, which means accepting that everything comes from Allah. Well, I’m practicing that fully." (P4).

“I do get upset at times, but then I pull myself together. I say, 'What Allah wills, will happen.’ We’re all destined to die someday. There’s no escaping that.” (P5).

“My faith, I never lost my faith, to be honest. Thank Allah. I prayed, I said 'My Lord.' Without Him, I couldn't have overcome this. First, you must seek refuge in your Lord, the rest follows naturally.” (P8).

“I continue living my life the same way. Today is like yesterday. I don't say, 'Oh, I'm going to pray a lot or do a lot of worship because I might die.' I haven't made any changes in my life.” (P9).

“Of course, I start by being thankful, thinking that I'm lucky. My diagnosis came about because my cousin's daughter passed away due to breast cancer. After her diagnosis, she passed away within two weeks. Following that, I got a mammogram, and that's how the disease was detected.” (P11).

### Theme 4: Future Hopes and Expectations

This theme consisted of the subthemes of “Future Thoughts” and “Recommendation for the Future.”

The first subtheme encompassed the concepts of innovation, uncertainty, and anxiety about the future. The participants expressed a desire for change in their lives, and they shared how uncertainty about the future had caused them to feel anxious. Some of the participants’ statements were as follows:

“I want to clear my head. I want to engage in activities that are focused on myself. I want to break free from this routine. Treatment of patients in hospitals needs to be improved. There should be more patience with patients and more attention should be paid to hygiene. Every patient should receive equal treatment. Compassion and dedication are crucial. Staffing levels should be increased, and there should be better collaboration.” (P1).

"When you start thinking about tomorrow, it can drive you crazy. You end up in a kind of depression, wondering what will happen tomorrow, what will happen? Don't say, 'I'll do this or that tomorrow.' Who knows if we'll even live to see tomorrow or if something unexpected will happen? Anything can happen; people have suddenly lost their lives in earthquakes. When you live in the moment, you've already invested in tomorrow. Everyone should cherish the present. They should realize the value of every second they are living. Whatever they are doing in that moment is the greatest gift. Finding joy in life is crucial. When you take care of your emotional well-being, you find happiness. Taking care of one's heart is even more important. How can you truly nourish it? Our Prophet used to pray, 'O Allah, grant me the joy of life.' Just think about it, our Prophet did that." (P2).

"I can't think about the future because nobody knows what might happen at any moment. Do I have thoughts about the future? Well, not really. We're all living under Allah's protection. Do I have any specific goals or objectives? My sole goal and purpose are to raise my child to be a good person for as long as I can." (P5).

“Recommendations for the Future” included concepts such as cherishing the present moment, nurturing emotional well-being, seeking psychological support, and the participants’ expectations of their healthcare providers. Some of the participants’ statements were as follows:

"Thankfully, I didn't get severely ill. I only experienced some dizziness, but people often feel devastated when they first receive the news. They don't know what to do afterwards. I believe that offering psychological counseling is essential in such cases. I think there should be a dedicated psychological support unit available." (P4).

"What I hope to experience at the hospital is just to see the nurses and staff members smiling. If there were a way for me to contribute, I’d also love to work there and devote myself to patients. Honestly, it's something I really want to do." (P5).

## Discussion

This study qualitatively examined the experiences of breast cancer patients as they made the transition from active treatment to survivorship. The research sheds light on significant themes related to the care that breast cancer patients encounter on their journey toward survival. Understanding how individuals perceive and experience breast cancer treatment and recovery will not only impact survival rates but also the overall healing process.

The study centered around four primary themes: (a) “a life changed by cancer and the difficulties encountered”; (b) “transition from active treatment to treatment-free living”; (c) “coping mechanisms”; and (d) “future hopes and expectations.” Drawing from these themes, the discussion can be structured under these four overarching headings.

- (a) A Life Changed by Cancer and the Difficulties Encountered

Breast cancer treatment and the post-treatment phase are marked by numerous challenges (Drageset et al., 2020; Zhai et al., 2019). Among these stages, the most prominent moments are often the initial reception of the diagnosis and concerns related to body image. Cancer treatment entails physical changes that affect women’s sense of femininity, fertility, self-esteem, and sexual functioning, placing them in a vulnerable position (Triberti et al., 2019). Following the interviews with the participants, it became evident that even though women who have survived breast cancer aspire to re-normalize their lives, they often realize that they can never fully live as they previously did.

The literature suggests that women who have experienced breast cancer undergo shifts in their philosophies of life, may embark on new life paths, alter their priorities, and generally develop a greater appreciation for life (Zhai et al., 2019). The individuals who participated in our study expressed their hopes for the future. In other studies that support the findings of the current research, cancer survivors have conveyed a sense of joy at having lived through the hardships of cancer, emphasizing how challenging it was to survive, while still maintaining a sense of gratitude (Ciria-Suarez et al., 2021; Santos et al., 2020).

The women in this study voiced their major concerns regarding changes in body image resulting from breast surgery and the experience of hair loss. Alopecia, in particular, is a profoundly emotional and traumatic condition that serves as an ongoing reminder of their battle with cancer. Hair loss, in this context, can significantly impact self-confidence and lead to a diminished sense of self-esteem (Daniel et al., 2021).

Breasts are widely perceived as a significant symbol of femininity and gender identity. Studies have indicated that women often find themselves feeling less attractive and experiencing disruptions in their body image following surgery. These changes have been noted to have a negative impact on their ability to cope with the illness (Mehrabi et al., 2016). Women, in particular, tend to experience a sense of loss, especially with regard to their relationships with their partners.

Many women express concerns about losing their breasts or experiencing negative changes in their appearance (Hamid et al., 2021). Another study also reported similar findings, suggesting that concerns about body image can lead to stress among survivors. In response to these concerns, women often rely on makeup, wigs, and breast reconstruction surgery to conceal any perceived physical imperfections (Hu et al., 2021; Zeighami Mohammadi et al., 2018).

The women in this study who had survived cancer consistently stated that financial difficulties were a major concern. The financial burden of cancer treatment extends not only to individuals diagnosed with cancer but also to their families and the broader society. In particular, the cost associated with esthetic and plastic surgical procedures represents an additional challenging aspect. Our findings align with a study conducted in Kashmir, which reported that women faced financial constraints, leading some to forgo esthetic treatments. Additionally, a portion of women experienced delays in treatment due to financial hardship (Hamid et al., 2021). Moreover, the cost of breast cancer treatment and rehabilitation poses a financial burden on survivors as well (Hu et al., 2021).

The concept of self-efficacy emerged as a prominent theme in the present study. With advancements in healthcare, cancer has become a treatable disease, highlighting the significance of developing self-efficacy skills that encompass lifestyle changes. Therefore, the period following a cancer diagnosis calls for individuals with breast cancer to reevaluate their priorities and embrace a healthier lifestyle with the aim of improving their health (Luo et al., 2021).

- (b) Transition from Active Treatment to Treatment-Free Living

The patients expressed their fears of their cancer recurring, and, in some cases, of death, now that their treatment process had concluded. Consistent with our research, another study found that women voiced concerns about the possibility of a recurrence of cancer (Fadhlaoui et al., 2021). Elmir et al. also noted that women with breast cancer experienced panic and insecurity due to the fear of cancer recurrence after treatment (Elmir et al., 2010). Insufficient information can indeed lead to anxiety, insecurity, and fear (Bigatti et al., 2023; Ciria-Suarez et al., 2021).

Consistent with these findings, the women experienced stress due to the physical symptoms of the disease and treatment side effects, fear of death, the progression of the disease, and the fear of what the future would bring. A study conducted in China reported that women also experience stress due to the fear of rejection by others because of the changes in their bodies and the possibility of cancer recurrence (Hu et al., 2021). Improving the self-efficacy and physical health of survivors are among the measures that need to be taken to mitigate the risk of recurrence (Chao et al., 2020).

- (c) Coping Mechanisms

The subthemes in this topic included factors that facilitated or hindered the transition to wellness, spirituality, and religious values and rituals. The participants discussed factors that helped or obstructed transition, such as acceptance, ignoring the illness, support, and emotional strength.

In the study, the women highlighted the importance of support from their spouses and families, and social support. Breast cancer treatment is an emotionally challenging time (Ciria-Suarez et al., 2021). The care and support of family members play a crucial role in patients' recovery, but sometimes excessive care can also cause stress (Hu et al., 2021).

A breast cancer diagnosis impacts women individually as well as those in their environment. The literature and the present study show that women often try to maintain a positive attitude during difficult times in order to protect their family's and especially their children’s well-being (Campbell-Enns & Woodgate, 2017). Family is a primary support source but not always effective, as family members may not fully understand the stress of living with cancer (Ciria-Suarez et al., 2021). The present study also emphasized spousal support. Patients may try to work together as a couple to share emotions, receive support, and overcome cancer-related problems (Luo et al., 2021).

Social support improves the health and quality of life in women with breast cancer. We found that support from different systems, such as the immediate family, other relatives, friends, and neighbors helped the women fight their illness. Support from spouses, families, close relatives, friends, and peer groups is crucial in adapting to breast cancer and maintaining hope for the future (Hamid et al., 2021; Mokhtari et al., 2022).

Most women in the present study had accepted their illness and continued their lives as usual. Studies have shown that women cope by continuing to practice their usual routines (Elmir et al., 2010; Hamid et al., 2021). Living and working as usual makes survivors feel like normal people and that they are in control (Hamid et al., 2021).

We found that spirituality and religious belief significantly influenced all the women participating in the present study. Spirituality is an important coping mechanism for women with breast cancer. It can provide comfort and peace in a way that is dynamic and unique to each individual. It instills hope and a positive outlook for the future. Spirituality and spiritual strength can enhance women’s beliefs in their ability to connect personally with a Supreme Being or “Creator” (Leão et al., 2021).

For some patients, spirituality is not only about finding new purposes in life but also about realizing them, while for others, it is inherently connected to religion. Belief in Allah is a source of hope, gratitude, and submission to divine will. The desire for life emerges from this search for meaning and valuing spiritual reality. Viewing life through a spiritual lens transforms ordinary experiences into something special, giving meaning to painful and challenging experiences (Leão et al., 2021).

Most women in the present study trusted their faith and Allah during the transition from illness to health, using religious practices like reading the scriptures and praying to cope with negative emotions like anxiety, anger, and depression. Some deepened their relationship with Allah (Leão et al., 2021).

In terms of coping mechanisms, all the women were reliant on religious practices and religious values to cope with their illness. This situation provided them with the power to accept reality while remaining hopeful that they would be healed (Hamid et al., 2021). The present study found that religious practices and religious faith helped the women to deal with their cancer. The women’s belief in Allah and fate, and their faith in their own destinies, were very strong.

They prayed, read the Quran, said prayers, and looked for ways to feel grateful about their situation in order to handle their pain and give a sense of shape and beauty to their lives. They submitted unconditionally to what they believed was Allah’s will and thus demonstrated that they had trust in Him. This is consistent with the findings in the literature (Tam Ashing et al., 2003). Religious belief is a significant source of support and strength in coping with the disease among women with breast cancer (Hamid et al., 2021).

A study on the experiences of African American women also revealed that spirituality and religion played a significant role in their survival rates during the process of coping with their illness (Adams et al., 2017). It is evident that most cancer survivors face specific challenges after completing their primary cancer treatment. However, when faced with these difficulties, cancer survivors in different countries can have varying experiences (Luo et al., 2021).

- (d) Future Hopes and Expectations

The final theme encompassed hopes and expectations for the future. The women expressed a desire to re-engage with life, while also experiencing anxiety and uncertainty about what might lie ahead.

The transition from illness to health can be seen as a journey of self-renewal, and nearly all survivors of breast cancer are actively trying to improve their health (Luo et al., 2021). Similarly, Rashidi and colleagues (2021) noted that individuals with breast cancer have to go through a process of “reconstructing identity” and ultimately developed a renewed sense of self in order to become stronger (Rashidi et al., 2021).

Meleis’ transition theory identifies five interrelated characteristics: awareness, engagement, change, difference, and time span (Meleis et al., 2000). During the transition to health, the most significant issue affecting women with breast cancer is “uncertainty” (Chao et al., 2020). It is important to remember that, according to Meleis (2010), a person in transition moves from a state of uncertainty to one of greater awareness.

The findings of the present study demonstrated that the women experienced uncertainty regarding their illness. However, it is also apparent that the feeling of uncertainty in the lives of those who have overcome breast cancer is not limited to this transition period alone. According to Meleis (2010), awareness and self-acceptance are the key concepts of transition. Individuals tend to develop coping strategies and new behaviors that facilitate transition (Meleis, 2010).

The women’s greatest concerns were uncertainties about their future, and worries related to their children and family, which is supported by the literature (Hamid et al., 2021; Mehrabi et al., 2016). In their statements about the future, the patients emphasized living in the moment, nurturing their emotional well-being, seeking psychological support, and their expectations of healthcare professionals. After being diagnosed with breast cancer, patients undergo various treatments. The most significant experiences during these phases, consistent with the literature, are the impact of side effects on the physical aspects of quality of life, as well as on psychological well-being (Ciria-Suarez et al., 2021).

Supporting the present study, despite many women experiencing psychological distress, only a few are referred to psychological services (Daniel et al., 2021). A qualitative study in Kashmir reported that women, despite experiencing trauma, often chose not to seek psychological counseling due to the stigma attached (Hamid et al., 2021).

In the present study, the women expressed a desire for more empathy and attention from healthcare professionals. This is supported by findings showing that survivors often feel they have not received adequate support from healthcare workers (Hu et al., 2021). Another study highlighted a lack of support and follow-up from nurses (Fadhlaoui et al., 2021). The role of healthcare professionals is crucial in reducing the psychological stress of survivors and aiding their rehabilitation (Hu et al., 2021).

Providing information and emotional and social support are critical for improving positive treatment outcomes, and both family members and health professionals are key sources of support (Adam & Koranteng, 2020). Healthcare workers should recognize the factors that facilitate and hinder the transition from illness to health and assist patients in this process (Chao et al., 2020).

### Limitations and Strengths of the Study

This study was carried out with Turkish breast cancer patients and its findings reflect the nuances of Turkish culture, traditions, and ways of life. Given this cultural specificity, certain aspects of the transition to health explored in this study might not fully resonate with the experiences of breast cancer patients in other countries.

## Conclusion

This study revealed a variety of themes which had a complex array of meanings in the lives of breast cancer survivors as they navigated their transition to health. It is evident that these survivors were influenced by numerous factors, with all participants using religious and spiritual practices as a way to cope. The results clearly demonstrate that these individuals were eager to put cancer behind them and return to a sense of normalcy in their lives, and that they felt a strong need to be supported in this journey. Their experiences revealed a series of factors that both facilitated and hindered their progress.

Understanding these factors during the transition period and providing support after treatment is crucial. Healthcare professionals and nurses should be aware of these factors that facilitate and hinder the transition to health, helping patients manage the challenges associated with it. The transition process for breast cancer survivors should be integrated into the curriculum for nursing students and the professional development programs for oncology nurses. This knowledge is vital in ensuring patients receive the best possible care. In this context, conducting comparative studies would be appropriate and beneficial in terms of understanding the broader scope of these experiences.
